# Radiomics of pericardial fat: a new frontier in heart failure discrimination and prediction

**DOI:** 10.1007/s00330-023-10311-0

**Published:** 2023-11-21

**Authors:** Liliana Szabo, Ahmed Salih, Esmeralda Ruiz Pujadas, Andrew Bard, Celeste McCracken, Maddalena Ardissino, Charalambos Antoniades, Hajnalka Vago, Pal Maurovich-Horvat, Bela Merkely, Stefan Neubauer, Karim Lekadir, Steffen E. Petersen, Zahra Raisi-Estabragh

**Affiliations:** 1 Semmelweis University, Heart and Vascular Center, Budapest, Hungary; 2 William Harvey Research Institute, NIHR Barts Biomedical Research Centre, Queen Mary University of London, Charterhouse Square, London, EC1M 6BQ UK; 3 Barts Heart Centre, St Bartholomew’s Hospital, Barts Health NHS Trust, West Smithfield, London, EC1A 7BE UK; 4 Departament de Matemàtiques I Informàtica, Universitat de Barcelona, Artificial Intelligence in Medicine Lab (BCN-AIM), Barcelona, Spain; 5 Division of Cardiovascular Medicine, Radcliffe Department of Medicine, University of Oxford, National Institute for Health Research Oxford Biomedical Research Centre, Oxford University Hospitals NHS Foundation Trust, Oxford, OX3 9DU UK; 6 National Heart and Lung Institute, Imperial College London, London, W12 0HS UK; 7 Royal Papworth Hospital, Papworth Rd, Trumpington, Cambridge, CB2 0AY UK; 8 Semmelweis University, Medical Imaging Centre, Department of Radiology, Budapest, Hungary; 9 Health Data Research UK, London, UK; 10 Alan Turing Institute, London, UK

**Keywords:** Machine learning, Magnetic resonance imaging, Pericardium, Adipose tissue, Radiomics

## Abstract

**Objectives:**

To use pericardial adipose tissue (PAT) radiomics phenotyping to differentiate existing and predict future heart failure (HF) cases in the UK Biobank.

**Methods:**

PAT segmentations were derived from cardiovascular magnetic resonance (CMR) studies using an automated quality-controlled model to define the region-of-interest for radiomics analysis. Prevalent (present at time of imaging) and incident (first occurrence after imaging) HF were ascertained using health record linkage. We created balanced cohorts of non-HF individuals for comparison. PyRadiomics was utilised to extract 104 radiomics features, of which 28 were chosen after excluding highly correlated ones (0.8). These features, plus sex and age, served as predictors in binary classification models trained separately to detect (1) prevalent and (2) incident HF. We tested seven modeling methods using tenfold nested cross-validation and examined feature importance with explainability methods.

**Results:**

We studied 1204 participants in total, 297 participants with prevalent (60 ± 7 years, 21% female) and 305 with incident (61 ± 6 years, 32% female) HF, and an equal number of non-HF comparators. We achieved good discriminative performance for both prevalent (voting classifier; AUC: 0.76; F1 score: 0.70) and incident (light gradient boosting machine: AUC: 0.74; F1 score: 0.68) HF. Our radiomics models showed marginally better performance compared to PAT area alone.

Increased PAT size (maximum 2D diameter in a given column or slice) and texture heterogeneity (sum entropy) were important features for prevalent and incident HF classification models.

**Conclusions:**

The amount and character of PAT discriminate individuals with prevalent HF and predict incidence of future HF.

**Clinical relevance statement:**

This study presents an innovative application of pericardial adipose tissue (PAT) radiomics phenotyping as a predictive tool for heart failure (HF), a major public health concern. By leveraging advanced machine learning methods, the research uncovers that the quantity and characteristics of PAT can be used to identify existing cases of HF and predict future occurrences. The enhanced performance of these radiomics models over PAT area alone supports the potential for better personalised care through earlier detection and prevention of HF.

**Key Points:**

*•PAT radiomics applied to CMR was used for the first time to derive binary machine learning classifiers to develop models for discrimination of prevalence and prediction of incident heart failure.*

*•Models using PAT area provided acceptable discrimination between cases of prevalent or incident heart failure and comparator groups.*

*•An increased PAT volume (increased diameter using shape features) and greater texture heterogeneity captured by radiomics texture features (increased sum entropy) can be used as an additional classifier marker for heart failure.*

**Supplementary information:**

The online version contains supplementary material available at 10.1007/s00330-023-10311-0.

## Introduction

Pericardial adipose tissue (PAT) is the visceral adipose tissue compartment surrounding the heart and coronary vasculature. An increasing body of evidence highlights associations between greater amounts of PAT and poorer cardiovascular outcomes [1–5]. Furthermore, higher PAT has been linked to adverse cardiovascular phenotypes, independent of multiple other measures of adiposity [6]. These associations are highly suggestive of a distinct mechanistic role of PAT in driving adverse cardiac remodelling, which are precursors of heart failure [6].

The mechanisms through which PAT influences myocardial structure and function are likely multifactorial, involving paracrine, vasocrine, and inflammatory pathways. PAT is known to secrete inflammatory factors and lipid metabolites [7], and this metabolic and secretory activity has been highlighted as an important factor driving adverse cardiovascular outcomes. At a cellular level, the secretome of PAT has been shown to adversely impact cardiomyocyte contractility [8], metabolism [9], and disrupt adhesion molecule expression in cardiac endothelial cells [10]. In the setting of ischemic heart disease, patterns of coronary atherosclerosis have been shown to closely follow superficial PAT distribution [11, 12]. Thus, existing evidence suggests that both the amount and character of PAT are important in determining its pathogenicity.

PAT can be quantified via CT and MRI cardiac imaging. Given the metabolic activity’s impact on myocardial structure and function, assessing tissue characteristics along with PAT volume might offer crucial insights into disease risk. Recent use of radiomics analysis methods on CT scans for characterising perivascular adipose tissue has greatly enhanced predictions of major cardiovascular events, outperforming traditional risk factors, coronary calcium scoring, coronary stenosis quantification, and high-risk plaque features [13]. Radiomics uses signal intensity (SI)–based data at a voxel level to provide quantitative information about patterns and distribution. Given that SI levels in cardiovascular magnetic resonance (CMR) reflect underlying tissue properties, it has been hypothesised that these metrics may reflect properties of the tissue from which they are extracted [14].

In previous work, we developed and validated an automated tool with in-built quality-control functions to allow extraction of PAT areas from over 40,000 UK Biobank CMR scans [15]. The availability of these data provides a key opportunity to further explore PAT character through radiomics analysis in a large-scale population-based cohort with rigorous prospective and retrospective clinical endpoint data. The aim of this study is to extend our work by performing deeper phenotyping of the pericardial adipose tissue character using radiomics analysis, to ascertain its value for classification and prediction of heart failure. This will involve extraction of radiomics phenotypes from PAT segmentation regions of interest, including information about the amount and geometry of PAT, as well as SI distribution and patterns. These phenotypes will then be used as predictor variables in machine learning models for (1) discriminating prevalent heart failure and (2) predicting incident heart failure. To aid clinical interpretation of the models, global and local explainability methods will be used to identify the key informative features from each model.

## Material and methods

**Fig. 1:**
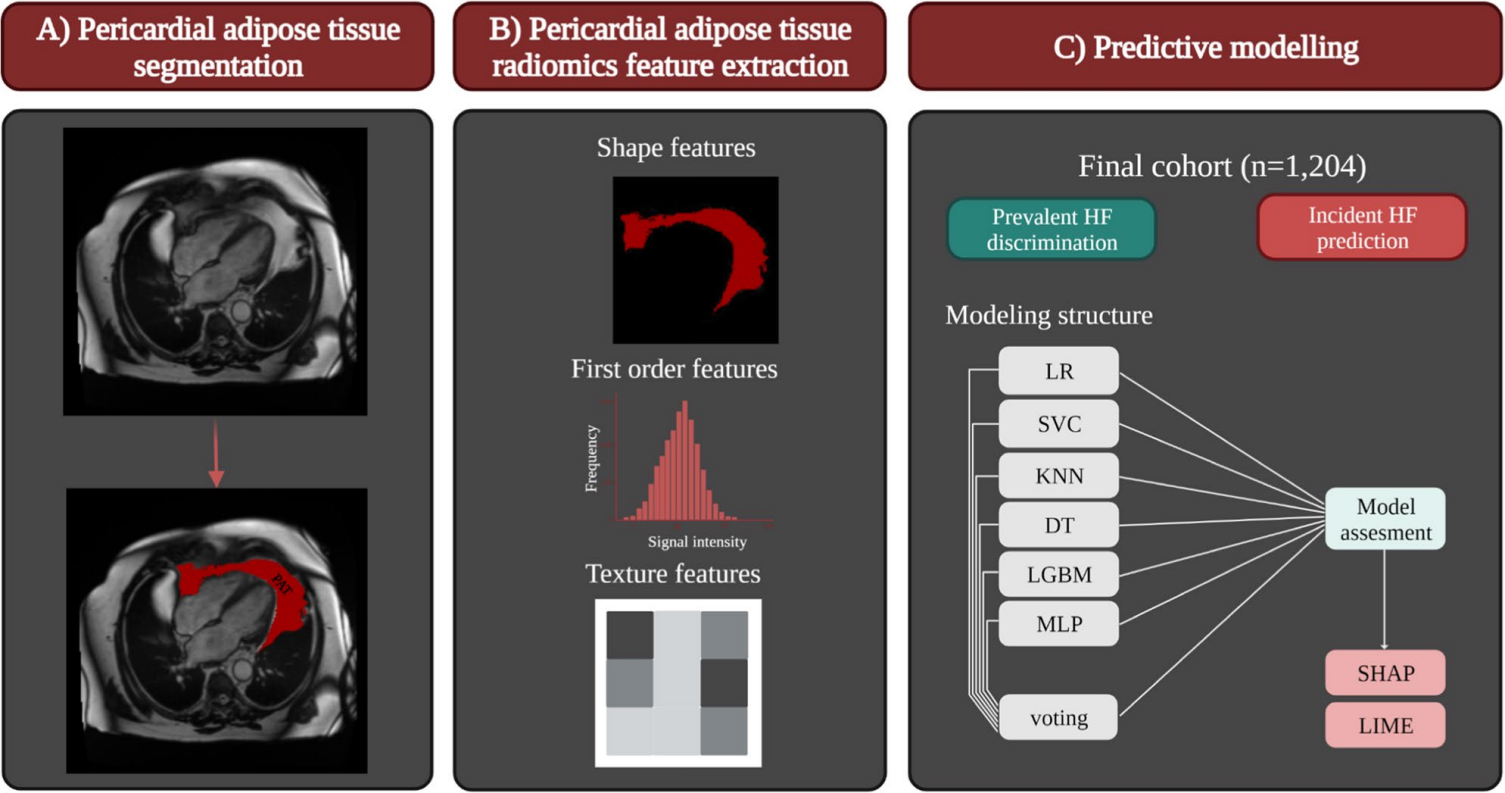
Schematic illustration of the study pipeline. **A** The segmentation of pericardial adipose tissue (PAT); color overlay represents the segmentation results as derived by Bard et al. **B** The radiomics feature extraction: PAT segmentation output was used to extract radiomics features from cardiovascular magnetic resonance imaging data. **C** The final cohort, which was assembled from prevalent and incident heart failure cases and randomly selected control individuals from the UK Biobank dataset

We provide a schematic illustration of the study pipeline in Fig. 1.

### Setting and study population

The UK Biobank is a cohort study incorporating more than 500,000 individuals from across the UK, aged 40–69 years old at recruitment between 2006 and 2010. Baseline assessment included socio-demographics, lifestyle, environmental factors, medical history, and a range of physical measures. Extensive electronic health record (EHR) linkages permit prospective tracking of health outcomes for all participants. The UK Biobank Imaging Study, which includes CMR, aims to scan a randomly selected 20% subset of the original participants.

### Ascertainment of heart failure status

HF status was ascertained using diseases codes from UK Biobank assessments and linked EHRs (Supplementary Table 1). Prevalent HF was defined as HF present at the time of imaging. Incident HF was considered as first occurrence of HF after imaging. The censor date was 30 September 2021 for incident HF outcomes giving an average follow-up of 3.7 ± 1.5 years from imaging.

### Definition of the comparator group

Participants with CMR available and no record of HF (prevalent or incident) were eligible for inclusion in the control group (*n* = 42,327). There was substantial imbalance between cases and eligible controls. Such imbalance results in poor model performance as the model prediction will be dominated by the majority class [16]. Given the extreme imbalance in our dataset, with a large number of controls compared to cases, and considering computational constraints we applied random undersampling to reduce the frequency of the controls relative to the cases. This approach randomly removes subjects from the majority classes to reach a final set of subjects in the majority class that are similar to the minority class. The final sample contained an equal number of randomly selected non-HF controls for both the prevalent and incident HF groups.

### Characterising the study sample

We accessed self-reported fields for participants’ educational level and smoking status. Material deprivation is reported as the Townsend index. Physical activity was measured via self-reported responses to the International Physical Activity Questionnaire. Diabetes, hypertension, and high cholesterol status were ascertained using information from self-report questions, physical measurements, and EHR data (Supplementary Table 2).

### Image acquisition

CMR scans were performed according to a pre-defined acquisition protocol using 1.5-Tesla scanners (MAGNETOM Aera, Syngo Platform VD13A, Siemens Healthcare) [17]. Cardiac function was assessed using standard long- and short-axis balanced steady-state free precession cine sequences.

### Extraction of pericardial fat segmentations

PAT segmentation was performed using an automated quality-controlled pipeline developed and validated in the UK Biobank and in an external cohort, as described by Bard et al [15]. In brief, PAT was measured from standard four-chamber cine images (single 2D slice) at phase 1 of the image cycle, which approximates end-diastole (ED). The contour was drawn to select areas of high signal intensity bordering the epicardial surface of the left and right ventricular myocardia. The ground truth manual segmentation was based on a sample of 500 randomly selected UK Biobank imaging sub-study participants using CVI42 post-processing software (Version 5.11, Circle Cardiovascular Imaging Inc.). Using the manual segmentation, a MultiResUNet neural network with Bayesian modification was trained for automated PAT segmentation with inbuilt quality control. Overall, the performance of the algorithm in test set relative to manual segmentations was good and very similar to the agreement between human observers (mean Dice score = 0.8) [15]. Automated PAT analysis was performed for all participants with adequate CMR imaging available (*n* = 42,929).

### Background to radiomics

Radiomics is an analysis technique permitting the computation of multiple descriptors of shape and texture [18]. The relevant information present in the image is extracted using three classes of features, namely (i) shape, (ii) first-order, and (iii) texture-based features. First-order features are histogram-based and relate to the distribution of the grey-level values in the tissue. Shape features capture the geometrical properties of the region of interest (ROI), including volume, diameter, minor/major axis, or sphericity. Texture features are derived from images using five matrices that encode the global texture information. They aim to describe patterns using mathematical formulae based on the spatial arrangement of pixels.

Lately, CMR radiomics features have been utilised to appreciate the heart’s complexity derived from the left and right ventricles, revealing patterns invisible to the naked eye [14]. There are as yet no existing reports of clinical models based on PAT radiomics features, likely due to the absence of appropriate datasets.

### PAT radiomics feature extraction

We used the PAT segmentation defined on the long-axis four-chamber images in the ED phase using the automated pipeline described above to derive our regions of interest (ROI) for radiomics analysis. We converted the contour points into binary masks, using a tool developed in-house, which we have made publicly available [19]. This software transformed delineated contour points for each ROI into a filled polygon in the coordinate space to form the binary mask. The harmonisation of the images was conducted using a histogram-matching technique applied to a reference image. The grey value discretisation was performed using a bin width of 25 to pull the intensity-based and texture radiomics features. The reference image for histogram matching was randomly selected, with careful consideration to ensure the chosen image did not contain artifacts. Histogram matching has been utilised effectively in prior radiomics-based models to standardise the intensity scale, thereby enhancing the model’s generalisation and classification capabilities [20–22]. In this study, imaging data was acquired using the same protocol (i.e., identical scanners and parameters) [17]. Therefore, we selected a single participant at random as the template for histogram harmonisation. This approach has been previously demonstrated to yield successful results in similar studies [20, 22]. The PyRadiomics platform (version 2.2.0) was adopted to extract 104 shape (*n* = 12), first-order (*n* = 17), and texture (*n* = 75) features from all PAT ROIs.

### Feature selection

To mitigate the risk of multicollinearity and increase the interpretability of our models, after feature extraction we performed a correlation analysis among radiomics features. Pairs of features exhibiting a correlation coefficient with an absolute value of 0.8 or above were identified. From each pair, we removed one feature to maintain the distinctiveness of the predictors in our model. Following this correlation-based feature selection process, we retained 28 features from the original 104. We also included age and sex in our model as they are known to significantly influence cardiac health. For comparative purposes, we also developed another model which incorporated overall PAT area, age, and sex as predictors.

### Predictive models

All the methods were implemented using Python version 3.9 and Scikit-learn [23] version 1.0.2. PAT radiomics features were used as predictors to classify prevalent HF and predict incident HF from non-HF controls. The features were normalised to zero mean and unit variance. We used seven binary classifiers followed by a voting classifier. We included the following classifiers to consider a wide variety of potential approaches: logistic regression (LR) [24], support vector classifier (SVC) [25], random forest (RF) [26], K-nearest neighbours (KNN) [27], decision tree (DT) [28], light gradient boosting machine (LGBM) [29], and multi-layer perceptron (MLP) [30]. To obtain each classifier’s optimal parameters, we used hyperparameter tuning and tenfold nested cross-validation, which consists of two loops [31]. The “inner” loop optimises model parameters using nine subsets for training and one for validation, repeating this process ten times to utilise every subset as a validation set once. The “outer” loop evaluates the optimised model on a separate test set, also iterated ten times.

Averaging these iterations provides an unbiased measure of the model’s ability to generalise to unseen data [32]. Supplementary Table 3 shows the parameters and their values of each model used to tune the parameters. The accuracy metric was used as a criterion to get optimal parameters from each model. Then the optimal parameters for each model were used to test the model. Accuracy, recall, precision, and F1 were used to assess the model performance within the test set. Finally, the voting ensemble was applied to combine and improve the performance of each individual prediction model using the hard voting approach. Voting classifier predicts the outcome based on the aggregation of the outcomes of each model. The following criteria were met to implement our voting classifier: (1) all classifiers produced reasonably good results; (2) all models within the ensemble generally already agree. Finally, we visualised the receiver operating characteristic (ROC) curves and area under the curve (AUC).

### Explainability methods

We used two explainability methods to highlight the most informative predictors in our machine learning models, including the magnitude and direction of their effect in relation to the outcome. The SHaply Additive exPlanations (SHAP) [33] method was used to interpret the model globally for all subjects. SHAP is a model agnostic method that can be applied to any model. It is based on game theory and reveals the effect of each predictor on the outcome. It calculates a score for each feature in the model which shows the size and direction effects of the feature on the outcome. In addition, we used Local Interpretable Model-Agnostic Explanations (LIME) [34] to explain the model locally for individual subjects. LIME is a local surrogate model that approximates the prediction of a given model. It does not train a global surrogate model, instead it trains a surrogate model locally for a subject. Thus, we applied both SHAP and LIME to explain the model globally and locally. For that purpose, we used the best performing classifier from each set of models to explain how the model works and predict an outcome for the test data.

## Results

### Description of baseline characteristics

**Fig. 2:**
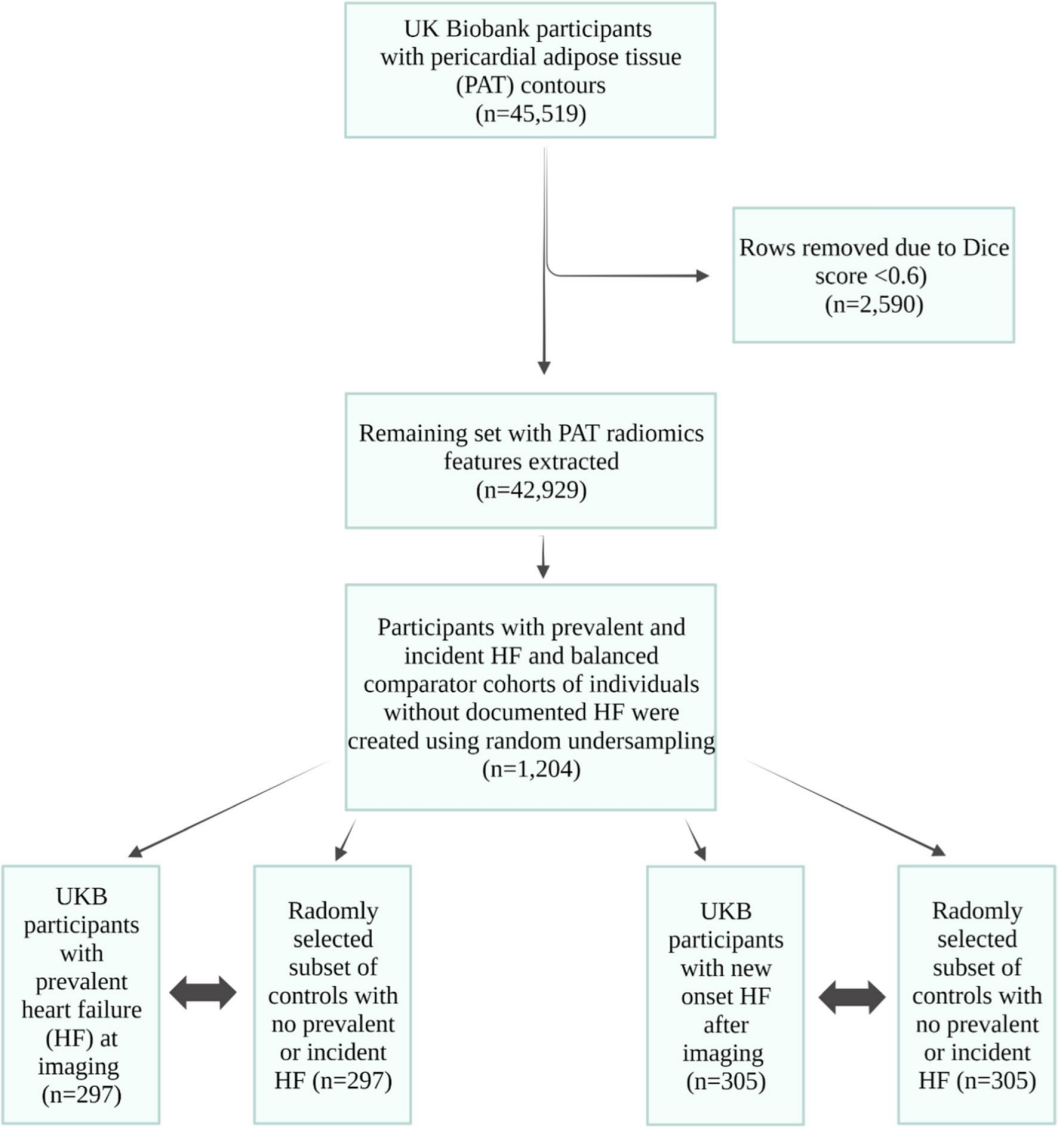
Study flowchart showing how final study sample size was derived. HF, heart failure; PAT, pericardial adipose tissue; UKB, UK Biobank

The imaging dataset was available for 43,226 UK Biobank participants (Fig. 2). Overall, 1204 participants from the UK Biobank Imaging Study were included in this study. The sample comprised 297 prevalent HF cases (60 ± 7 years, 21% female) and 305 cases of incident HF (61 ± 6 years, 32% female), with randomly selected non-HF controls of similar size for each group. Baseline characteristics and risk factors for each group are depicted in Table 1. Generally, randomly selected non-HF controls were younger, and more likely to be female and well-educated. They had less deprivation and overall lower burden of risk factors compared to the diseased groups. Fewer participants with diabetes, hypertension, and high cholesterol were present in the prevalent HF comparator group than the disease group. In contrast, the incident HF control group had an equal number of participants with these risk factors.

**Table 1:** Baseline characteristics

Characteristics	Prevalent HF group (*n* = 297)	Prevalent HF control group (*n* = 297)	Incident HF group (*n* = 305)	Incident HF control group (*n* = 305)
Age, years (± SD)	59.9 (± 6.7)	55.2 (± 7.5)	61.11 (± 6.3)	54.94 (± 7.3
Female sex, *n* [%]	63 [21.4%]	165 [55.9%]	95 [32.3%]	152 [50.3%]
Townsend deprivation index	− 1.2 (± 3.2)	− 2 (± 2.8)	− 1.7 (± 2.8)	− 1.81 (± 2.7)
Post-secondary education, *n* [%]				
• None	40 [13.8%]	11 [3.9%]	45 [15.2%]	16 [5.4%]
• CSE or equivalent	12 [4.2%]	14 [4.8%]	4 [1.7%]	13 [4.4%]
• O levels/GCSEs or equivalent	59 [20.4%]	58 [19.9%]	72 [24.3%]	62 [20.9%]
• NVQ or HND or HNC or equivalent	31 [10.7%]	19 [6.5%]	22 [7.4%]	9 [3%]
• Other professional qualifications	20 [6.9%]	8 [2.8%]	10 [3.4%]	19 [6.4%]
• A levels/AS levels or equivalent	33 [11.4%]	36 [12.4%]	35 [11.8%]	29 [9.8%]
• College or university degree	94 [32.5%]	145 [49.8%]	107 [36.2%]	149 [50.2%]
BMI, kg/m^2^ (± SD)	28.7 (± 5)	26.4 (± 4.1)	28 (± 4.9)	26.30 (± 4.1)
Physical activity, summed MET-min/week (± SD)	2038 (± 2127)	2646 (± 2514)	2329 (± 2281)	2421 (± 2225)
Current smoker, *n* [%]	11 [3.7%]	8 [2.7%]	12 [4%]	14 [4.6%]
Diabetes status, *n* [%]	65 [22%]	13 [4.4%]	43 [14.1%]	21 [7%]
Hypertension status, *n* [%]	251 [85.1%]	103 [34.9%]	104 [34.2%]	110 [36.4%]
High cholesterol status, *n* [%]	233 [79%]	101 [34.2%]	125 [41.1%]	123 [40.7%]

Counts variables are presented as number [percentage] of non-missing values; continuous variables as mean (standard deviation)

*Abbreviations*: *BMI* body mass index, *CSE* Certificate of Secondary Education, *GCSE* General Certificate of Secondary Education, *HND* Higher National Diploma, *HNC* Higher National Certificate, *MET* metabolic equivalent of task, *NVQ* National Vocational Qualification

### Prevalent heart failure prediction using pericardial adipose tissue radiomics

**Fig. 3:**
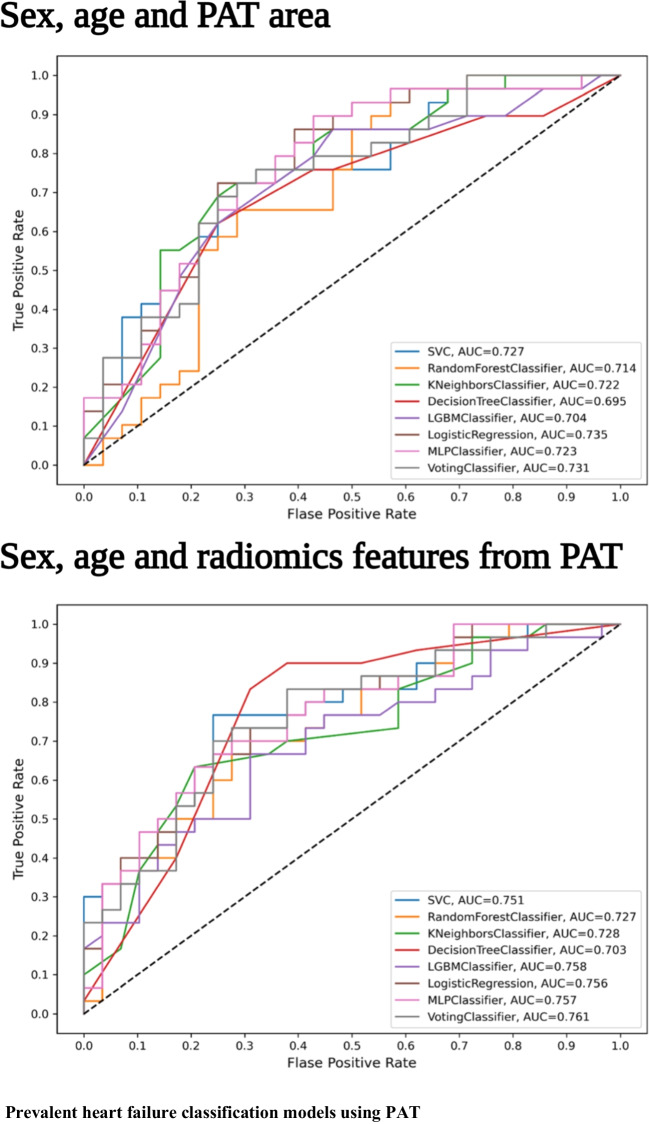
The first panel shows the results from prevalent heart failure classification using sex, age and PAT area. The second panel illustrates the results from prevalent heart failure classification using sex, age and radiomics features derived from PAT

The classification models, incorporating radiomics features along with sex and age, demonstrated good discrimination between participants with prevalent HF and controls (Table 2, Fig. 3). The voting classifier method, among the tested models, exhibited the highest discriminative power, achieving an AUC of 0.76 and an F1 score of 0.70 in our test set. Consistency in performance was observed across various models including SVC, KNN, RF, LGBM, and the voting classifier, while DT, LR, and MLP models showed slightly lower discriminative power. However, significant performance variation was only observed between the DT and LR models (Supplementary Table 4). Our radiomics feature-based models consistently showed slightly better performance compared to models using mean PAT area as the predictor (voting classifier AUC: 0.73).

**Table 2:** Model output for prevalent HF classification

Model	AUC	Accuracy	F1	Recall	Precision
Support vector machine	0.751 (± 0.05)	0.695	0.688	0.682	0.697
Random forest	0.727 (± 0.05)	0.676	0.670	0.665	0.680
K-nearest neighbour	0.728 (± 0.04)	0.668	0.668	0.734	0.650
Decision tree	0.703 (± 0.06)	0.678	0.711	0.789	0.665
LightGBM	0.758 (± 0.06)	0.683	0.673	0.658	0.692
Logistic regression	0.756 (± 0.05)	0.697	0.690	0.682	0.699
Multi-layer perceptron	0.757 (± 0.05)	0.703	0.699	0.700	0.704
Voting	0.761 (± 0.05)	0.707	0.704	0.706	0.705

The models’ performance for prevalent heart failure discrimination for each classification method applied. Voting classifier predicts the outcome based on the aggregation of the outcomes of each individual binary classification model

*Abbreviations*: *LR* logistic regression, *SVC* support vector classifier, *RF* random forest, *KNN* K-nearest neighbours, *DT* decision tree, *LGBM* light gradient boosting machine, *MLP* multi-layer perceptron

**Fig. 4:**
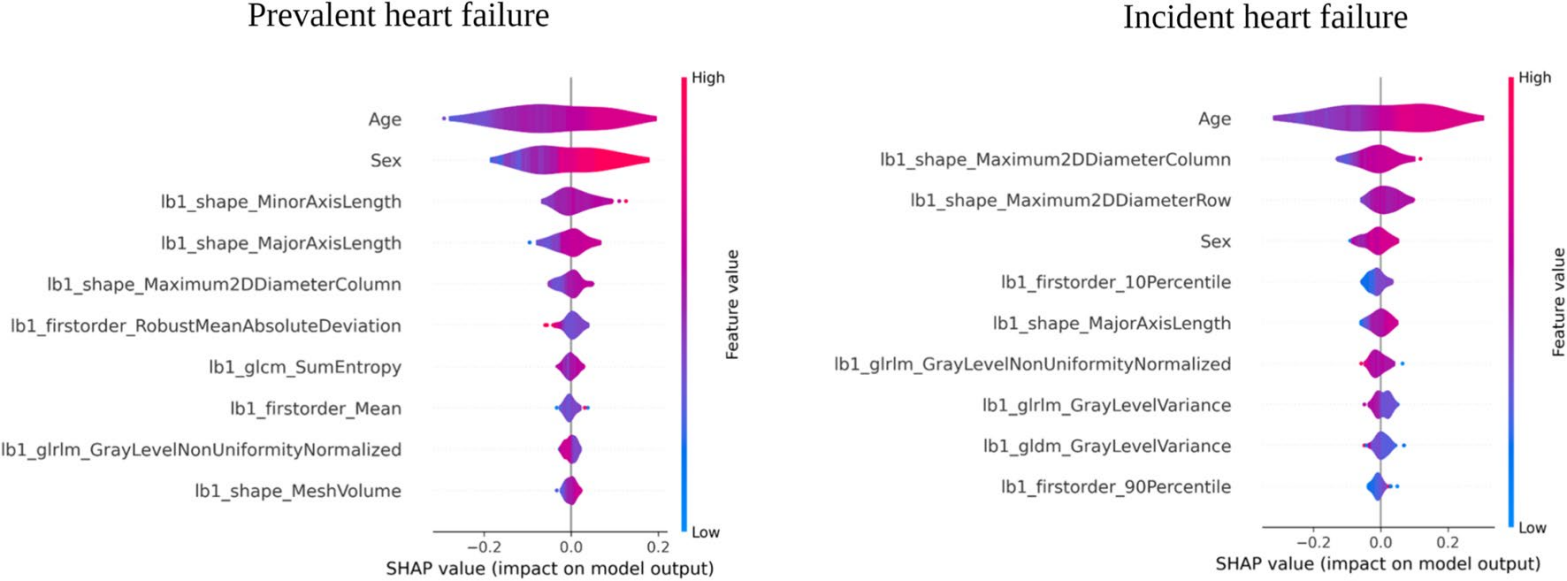
Ten most informative predictors based on the Shapley explainability models. The beeswarm plots show the ten most informative predictors from the voting classifier for each prevalent heart failure and from the LGBM classifier for incident heart failure prediction in order of decreasing feature importance. The y-axis represents the name of the features chosen by the model as predictors, while the x-axis indicates the contribution of each feature to the outcome. Each dot represents a subject and the color indicates the feature value. For instance, the increasing the value of the first feature (Minor Axis Length) would lead to and increased probability of a subject having prevalent heart failure at the time of CMR imaging

**Fig. 5:**
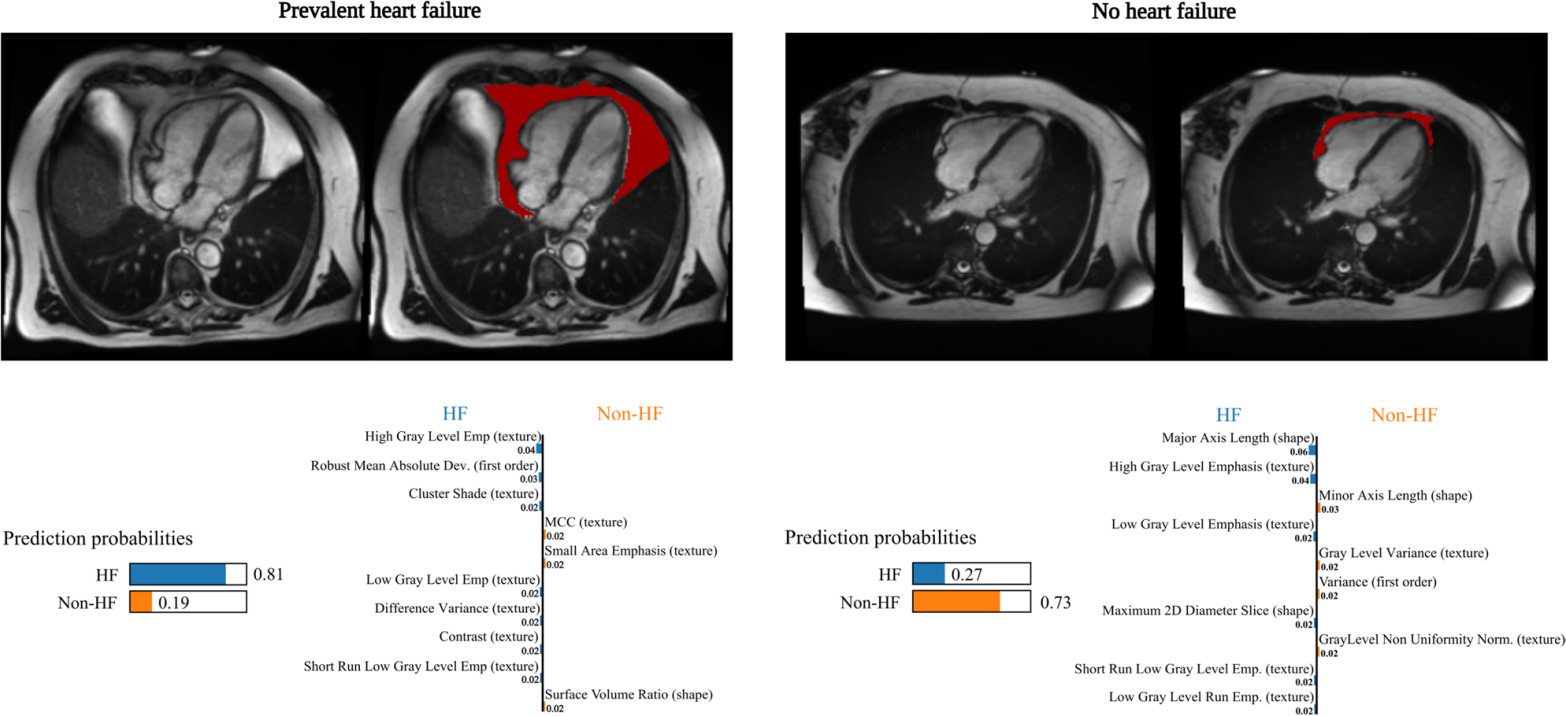
Two cases illustrating the output from the LIME explainability models. Output from LIME explainability models for two subjects in our dataset. In the first case where the ground truth labeling is “prevalent heart failure”, the model is 81% certain that the subject is indeed a prevalent hear failure patient, while still showing a 19% probability that the case could be a control. The second plot shows that the model is 73% certain that the subject is from the control group, which is indeed true based on the ground truth labeling. For both subjects, the figure shows the contribution of each radiomics feature. Moreover, the numerical value beside each feature shows the magnitude effect of each feature in each class

To interpret feature importance, we employed the SHAP, focusing on the results from the voting classifier model (Fig. 4). According to the SHAP analysis, sex and age were the most influential features. Among the shape features, minor axis length and major axis length, which describe the overall amount of pericardial fat, were highlighted as important. Regarding texture features, robust mean absolute deviation (statistical measure of the dispersion of intensity values) and GLCM sum entropy (measure of the complexity of the distribution of paired pixel intensities) were identified as key metrics differentiating prevalent HF from controls. The LIME explainability model demonstrates our model’s performance, highlighting instances of correct classifications in Fig. 5, and misclassifications in Supplementary Fig. 1.

### Incident heart failure prediction using pericardial adipose tissue radiomics

**Fig. 6:**
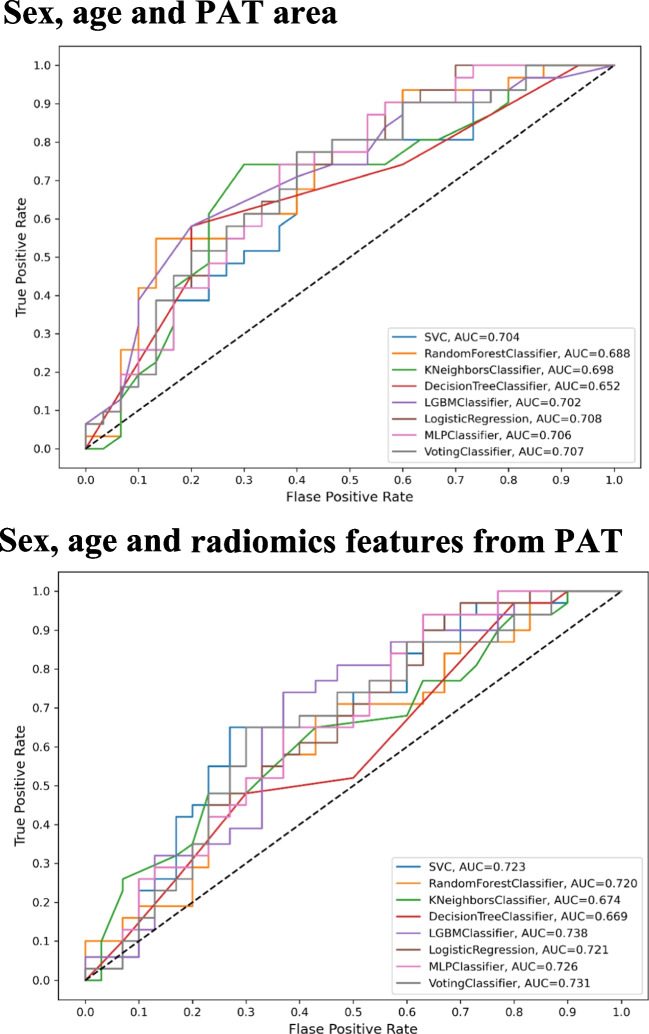
Incident heart failure prediction models using PAT. The first panel shows the results from incident heart failure prediction using sex, age, and PAT area. The second panel illustrates the results from incident heart failure prediction using sex, age, and radiomics features derived from PAT

Our models for incident HF prediction demonstrated slightly lower performance as compared to those for prevalent HF classification. The model utilising the LGBM classifier emerged as the most effective for incident HF prediction, attaining an AUC of 0.74 and an F1 score of 0.68 (Table 3, Fig. 6). Despite some minor differences, overall model performance remained relatively stable across the various classifiers. Although the KNN and DT classifiers exhibited marginally lower AUC and F1 scores, these differences did not achieve statistical significance between any two methods (Supplementary Table 5). Predictive models using PAT area alone reached lower predictive values compared to our radiomics models across all ML methods (LGBM classifier AUC 0.71).

**Table 3:** Model output for incident HF prediction

Model	AUC	Accuracy	F1	Recall	Precision
Support vector machine	0.723 (± 0.07)	0.651	0.646	0.640	0.656
Random forest	0.720 (± 0.07)	0.672	0.673	0.679	0.672
K-nearest neighbour	0.674 (± 0.09)	0.630	0.611	0.584	0.643
Decision tree	0.669 (± 0.07)	0.634	0.625	0.623	0.639
LightGBM	0.738 (± 0.06)	0.684	0.682	0.685	0.683
Logistic regression	0.721 (± 0.07)	0.649	0.645	0.639	0.654
Multi-layer perceptron	0.726 (± 0.07)	0.656	0.650	0.642	0.662
Voting	0.731 (± 0.07)	0.679	0.678	0.675	0.681

The models’ performance for incident heart failure prediction for each classification method applied. Voting classifier predicts the outcome based on the aggregation of the outcomes of each individual binary classification model

*Abbreviations*: *LR* logistic regression, *SVC* support vector classifier, *RF* random forest, *KNN* K-nearest neighbours, *DT* decision tree, *LGBM* light gradient boosting machine, *MLP* multi-layer perceptron

Based on the SHAP analysis performed on the LGBM classifier, age emerged as the most influential predictor (Fig. 4). It was followed by size measures, with shape features such as increased maximum 2D diameter column and maximum 2D diameter row (indicative of elevated PAT value) serving as significant predictors of incident HF. Following size measures, sex was determined to be the next important predictor. The texture features, specifically the first-order ten percentile and GLRLM grey-level non-uniformity, were found to be important, but less influential when compared to the preceding predictors.

## Discussion

In this proof-of concept study, we set up a pipeline using radiomics feature extraction and machine learning to predict high-risk PAT phenotypes among UK Biobank participants undergoing CMR. We demonstrate for the first time that PAT radiomics can be used to discriminate prevalent HF cases from controls and predict incident HF. We found that shape and texture features showing an increased PAT volume and greater texture heterogeneity captured by radiomics can be used as an additional classifier marker for HF.

Interestingly, our study observed a significant difference in the prevalence of key risk factors such as diabetes, hypertension, and high cholesterol between the comparator group and the prevalent HF group. In contrast, the incident HF group had an equal distribution of participants with these risk factors, which is a direct result of our random undersampling approach, rather than indicative of differing risk profiles. Despite these disparities in risk factor prevalence, the classification models for existing HF performed only slightly better than those for predicting new-onset disease. This underscores the robustness of our models in handling variable distributions of risk factors and highlights the potential applicability of our approach in diverse clinical settings where the presence and distribution of comorbidities may vary significantly.

SHAP outputs based on the LGBM classifier illustrated that shape features showing greater PAT volume were the key predictors of incident HF in our study. This finding is in line with the results from Kenchaiah et al [5] who have demonstrated the pericardial fat volume was associated with an increased risk of HF in the Multi-ethnic Study of Atherosclerosis (MESA) cohort. Our results are additionally consistent with previous work from the UK Biobank, demonstrating association of greater PAT area with adverse cardiovascular phenotypes [6]. We significantly extend these existing observations by demonstrating the additional importance of PAT character in defining both prevalent and incident HF. The overall amount of pericardial fat captured either by PAT area or radiomics descriptors of shape helped explain the majority of differences between prevalent HF patients and comparator group. Furthermore, our explainability model applied at the output of the voting classifier showed that texture features capturing the greater local entropy of intensity values suggesting greater tissue heterogeneity within the pericardial fat were also dominant features in prevalent disease discrimination. This suggests that local SI heterogeneity, which might reflect on the fat tissue properties, is an important distinguishing feature of HF patients. Our findings support mechanistic research and highlight the importance of both amount and character of PAT in their potential to drive adverse cardiovascular complications.

The noted association between PAT and risk of HF has multiple potential explanations. First, it might relate to localised, long-term exposure of the myocardium to an inflammatory milieu. Deposition of PAT has been shown to be bidirectionally related to inflammation, and the extent of inflammation resultant from PAT deposition might be a key determinant of subsequent cardiovascular disease. Radiomics characterisation of PAT might thus improve the distinction between relatively “quiescent” PAT stores from the more “inflammatory” ones. Indeed, individuals with chronic inflammatory conditions are known to deposit more PAT relative to whole body fat mass when compared to controls [35]. On the other hand, PAT itself is also known to be highly metabolically active, and pre-clinical studies have demonstrated that it secretes multiple cytokines and inflammatory mediators that might contribute to a local, paracrine inflammatory effect [36]. To support this hypothesis, asymmetric, localised pockets of PAT have been noted to follow the distribution of coronary artery disease (CAD) in previous studies [11, 12, 37]. This suggests that either CAD or the resultant localised inflammation increases PAT deposition, or vice versa. This localised proinflammatory milieu contributing to regional inflammation would reasonably promote localised myocardial damage, which can contribute to myocardial dysfunction and HF [1, 7]. Second, the association between PAT and HF might relate to the widely recognised phenomenon of intracellular steatosis. Previous studies have identified an association between myocardial triglyceride content and cardiovascular events including heart failure hospitalisations [38]. To support this hypothesis, a previous investigation on the UK Biobank cohort identified triglycerides as major mediators of the association between PAT and adverse left ventricular measures on CMR [6].

Radiomics has been increasingly used and validated to improve the diagnostic and prognostic accuracy of medical imaging. Within CMR radiomics, features derived from the ventricular shape and myocardial tissue have been applied to the discrimination of ischaemic heart disease [39–41] and different cardiomyopathies [42–44]. Pericardial fat radiomics represents an additional layer of information we can derive from standard of care CMR scans. Critically, our results demonstrate that radiomics can be used to discriminate HF cases from controls, signifying a potential novel avenue for better diagnostic and prognostic assessment.

### Limitations

Our study provides initial insights into PAT radiomics for predicting HF though it has some important limitations. The models are preliminary and need further independent external validation. We used random undersampling due to extreme dataset imbalance, potentially leading to bias and overoptimistic performance estimates. While our models used a range of radiomics features and demographic variables, other relevant factors were not included, warranting comprehensive patient information in future research to enhance model performance.

## Conclusions

Machine learning classifiers built upon radiomics features depicting the amount (larger PAT diameters) and texture character (greater tissue heterogeneity) of pericardial fat can be used to discriminate individuals with prevalent heart failure and predict incidence of future heart failure.

### Supplementary information

Below is the link to the electronic supplementary material.

Supplementary file1 (PDF 333 KB)

## Abbreviations

CAD: Coronary artery disease
CMR: Cardiovascular magnetic resonance
DT: Decision tree
HF: Heart failure
KNN: K-nearest neighbours
LGBM: Light gradient boosting machine
LIME: Local interpretable model-agnostic explanations
LR: Logistic regression
MLP: Multi-layer perceptron
PAT: Pericardial adipose tissue
RF: Random forest
ROC: Receiver operating characteristic curves
SHAP: SHaply Additive exPlanations
SI: Signal intensity
SVC: Support vector classifier

## Declarations

### Guarantor

The scientific guarantor of this publication is Prof. Steffen Petersen.

### Statistics and biometry

AS and CM have significant statistical expertise and extensive experience working with UK Biobank data. AS led analysis and model development steps. CM led the creation of diagnostic labels, and performed dataset creation for all non-radiomic features.

### Study subjects or cohorts overlap

This research was conducted using the UK Biobank resource under access application 2964. UK Biobank will make the data available to all bona fide researchers for all types of health-related research that is in the public interest, without preferential or exclusive access for any persons. All researchers will be subject to the same application process and approval criteria as specified by UK Biobank. For more details on the access procedure, see the UK Biobank website: http://www.ukbiobank.ac.uk/register-apply. Automated, quality-controlled pericardial adipose tissue segmentation was conducted in a previous publication by our research group (Bard et al, https://www.frontiersin.org/articles/10.3389/fcvm.2021.677574/full). This article is referenced in the manuscript. Of note, the current study is not an extension but rather a completely new layer of the research conducted using pericardial adipose tissue analysis. The analysis and model development of this study were conducted independently.

### Methodology

•observational

•multicentre study
