# Supplementary material for: Radiomics of pericardial fat: a new frontier in heart failure discrimination and prediction
Source: Eur Radiol. 2023 Nov 21;34(6):4113–26. doi: 10.1007/s00330-023-10311-0 (PMC11166856; doi:10.1007/s00330-023-10311-0)

Supplementary Table 1. UK Biobank fields/codes for heart failure

Source	Field ID or code	Description
Heart failure		
Self-report	20002	heart failure/pulmonary odema
ICD9	428	428 Heart failure
ICD9	4020	4020 Hypertensive heart disease, specified as malignant
ICD9	4029	4029 Hypertensive heart disease, not specified as malignant or benign
ICD9	4040	4040 Hypertensive heart and renal disease, specified as malignant
ICD9	4049	4049 Hypertensive heart and renal disease, not specified as malignant or benign
ICD10	I110	I11.0 Hypertensive heart disease with (congestive) heart failure
ICD10	I130	I13.0 Hypertensive heart and renal disease with (congestive) heart failure
ICD10	I132	I13.2 Hypertensive heart and renal disease with both (congestive) heart failure and renal failure
ICD10	I500	I50.0 Congestive heart failure
ICD10	I501	I50.1 Left ventricular failure
ICD10	I509	I50.9 Heart failure, unspecified
First occurrences	131354	Date I50 first reported (heart failure)

Supplementary Table 1 footnote. ICD10 codes are drawn from fields 41270, 41280, 41234 and 41259; ICD9 codes are drawn from fields 41271, 41281, 41234 and 41259

Supplementary Table 2. UK Biobank fields/codes for risk factors

Source	Field ID or code	Description
Diabetes		
Self-report	20002	Diabetes
	20002	Type 1 diabetes
	20002	Type 2 diabetes
Medications	6153, 6177: 3	Insulin
ICD9	250	Diabetes mellitus
ICD10	E10	E10 Insulin-dependent diabetes mellitus
ICD10	E11	E11 Non-insulin-dependent diabetes mellitus
ICD10	E12	E12 Malnutrition-related diabetes mellitus
ICD10	E13	E13 Other specified diabetes mellitus
ICD10	E14	E14 Unspecified diabetes mellitus
ICD10	O24	O24 Diabetes mellitus in pregnancy
ICD10	R73	R73 Elevated blood glucose level
First occurrences	130706	Date E10 first reported (insulin-dependent diabetes mellitus)
First occurrences	130708	Date E11 first reported (non-insulin-dependent diabetes mellitus)
First occurrences	130710	Date E12 first reported (malnutrition-related diabetes mellitus)
First occurrences	130712	Date E13 first reported (other specified diabetes mellitus)
First occurrences	130714	Date E14 first reported (unspecified diabetes mellitus)
Diagnosed by doctor	2443	Diabetes diagnosed by doctor
Diagnosed by doctor	2976	Age diabetes diagnosed by doctor
Biochemistry	30740	Serum glucose >11.1 mmol/L
Biochemistry	30750	Glycated haemoglobin (HbA1c) > 48
Hypertension		
Self-report	20002	Essential hypertension
	20002	Hypertension
Medications	6153, 6177: 2	Blood pressure medication
ICD10	I10	Essential (primary) hypertension
First occurrences	131286	Date I10 first reported (essential (primary) hypertension)
Diagnosed by doctor	6150: 4	High blood pressure
	2966	Age high blood pressure diagnosed
High cholesterol		
Self-report	20002	high cholesterol
Medications	6153, 6177: 1	Cholesterol lowering medication
ICD10	E780	E78.0 Pure hypercholesterolaemia
ICD10	E781	E78.1 Pure hyperglyceridaemia
ICD10	E782	E78.2 Mixed hyperlipidaemia
ICD10	E783	E78.3 Hyperchylomicronaemia
ICD10	E784	E78.4 Other hyperlipidaemia
ICD10	E785	E78.5 Hyperlipidaemia, unspecified
First occurrences	130814	Date E78 first reported (disorders of lipoprotein metabolism and other lipidaemias)
Biochemistry	30690	serum total cholesterol >7 mmol/L

Supplementary Table 2 footnote:
ICD10 codes are drawn from fields 41270, 41280, 41234 and 41259; ICD9 codes are drawn from fields 41271, 41281, 41234 and 41259; Where a 3-digit code is given, this includes all 4-digit sub-codes, for example, E10 includes E100, E101 and E102

Supplementary Table 3: The parameters and values of each model used in hyperparameter tuning

Model	Parameters	Values
Logistic Regression (LR)	Regularization (C)	0.1, 1 ,10, 50, 100
Support Vector Classifier (SVC)	Regularization (C)	0.1,1 ,10, 50, 100, 120, 150, 200, 300
	Gamma	10, 5, 3, 1, 0.1, 0.01, 0.001
	Kernel	rbf, linear, poly
	Cache size	5, 10, 25, 50, 100, 200, 300, 400
Random Forest (RF)	Number of estimators	100 to 1000, increase by 200
	Max features	auto, sqrt
	Min samples leaf	1, 3, 5, 7
	Min samples split	2, 5, 10, 15
	Max depth	10 to 100, increase by 10
K-nearest Neighbours (KNN)	Number of neighbours	2, 5, 7, 9, 11, 13, 15, 30, 50
	Algorithm	ball_tree, kd_tree, brute, auto
	Weights	uniform, distance
	Metric	minkowski, euclidean, manhattan
	Leaf size	10, 30, 50, 70, 100, 200
Decision Tree (DT)	Max depth	2, 3, 5, 10, 20, 50
	Splitter	best, random
	Min samples leaf	5, 10, 20, 50,100
	Criterion	gini, entropy
	Min samples split	5, 10, 30, 50
Light Gradient Boosting Machine (LGBM)	Max bin	50, 100, 150, 200, 250, 300
	Boosting type	gbdt, dart, goss, rf
	Number of leaves	5,10,15,20,30
	Min child samples	5, 10, 20, 30, 40
	Learning rate	0.1, 0.01, 0.001, 0.2, 0.02, 0.002, 0.3, 0.03, 0.003
Multi-layer Perceptron (MLP)	Hidden layer sizes	(100,50,25, 12, 6, 3, 1), (150,100,50, 1), (100,100, 100, 1)
	Activation	tanh, relu, identity
	Solver	sgd, adam, lbfgs
	Alpha	0.0001, 0.001 ,0.05
	Learning rate	constant, adaptive

Supplementary Table 3 footnote: We used seven binary classifiers for modeling: Logistic Regression, Support Vector Classifier, Random Forest, K-nearest Neighbours, Decision Tree, Light Gradient Boosting Machine, and Multi-layer Perceptron. We applied hyperparameter tuning to reach the optimal parameters for each classifier using 10-fold cross validation. The parameters applied for each classifier are shown in the table.

Supplementary Table 4: Comparison between binary classification models of prevalent heart failure cases

Model	SVC	RF	KNN	DT	LGBM	LR	ML
RF	0.28						
KNN	0.51	0.54					
DT	0.06	0.19	0.13				
LGBM	0.38	0.50	0.38	0.19			
LR	0.62	0.44	0.61	0.03*	0.40		
ML	0.57	0.64	0.54	0.19	0.53	0.61	
Voting	0.46	0.38	0.66	0.05	0.43	0.43	0.49

Footnote: The table represents the paired T test p-values as per Dietterich et al[1] implemented using paired_ttest_5x2cv: 5x2cv paired *t* test for classifier comparisons - mlxtend (rasbt.github.io). Asterix signify statistically significant difference.

1: Thomas G. Dietterich; Approximate Statistical Tests for Comparing Supervised Classification Learning Algorithms. Neural Comput 1998; 10 (7): 1895–1923. doi: https://doi.org/10.1162/089976698300017197

Supplementary Table 5: Comparison between binary classification models of incident heart failure cases

Model	SVC	RF	KNN	DT	LGBM	LR	ML
RF	0.37						
KNN	0.59	0.51					
DT	0.16	0.09	0.30				
LGBM	0.32	0.49	0.39	0.40			
LR	0.31	0.50	0.52	0.12	0.47		
ML	0.47	0.41	0.52	0.34	0.50	0.59	
Voting	0.36	0.37	0.45	0.17	0.42	0.60	0.53

Footnote: The table represents the paired T test p-values as per Dietterich et al[1] implemented using paired_ttest_5x2cv: 5x2cv paired *t* test for classifier comparisons - mlxtend (rasbt.github.io).

1: Thomas G. Dietterich; Approximate Statistical Tests for Comparing Supervised Classification Learning Algorithms. Neural Comput 1998; 10 (7): 1895–1923. doi: https://doi.org/10.1162/089976698300017197

Supplementary Figure 1: Examples of cases where the model failed

Prevalent HF case classified as control

Control classified as prevalent HF

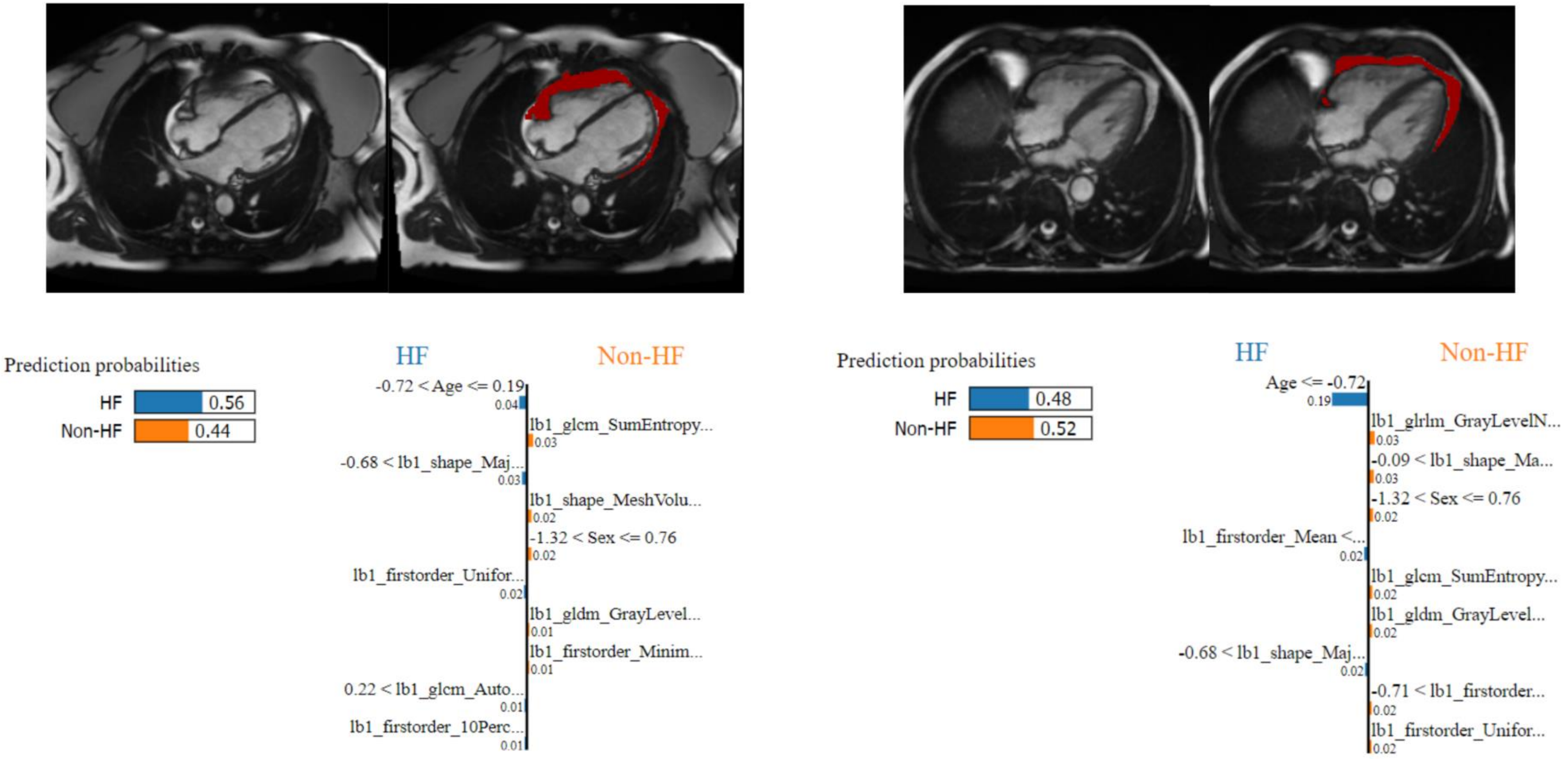

Supplement: Supplementary file 1 — Supplementary file1 (PDF 333 KB) [file 330_2023_10311_MOESM1_ESM.pdf]
